# Biological Activity of Recombinant Accessory Cholerae Enterotoxin (Ace) on Rabbit Ileal Loops and Antibacterial Assay

**Published:** 2012-12-12

**Authors:** Shaghayegh Anvari, Shahin Najar Peerayeh, Mehrdad Behmanesh, Mina Boustanshenas

**Affiliations:** 1. Department of Bacteriology, Faculty of Medical Sciences, Tarbiat Modares University, Tehran, Iran; 2. Department of Genetics, Faculty of Basic Sciences, Tarbiat Modares University, Tehran, Iran; 3. Science and Research Branch, Islamic Azad University, Tehran, Iran

**Keywords:** Vibrio cholerae, Accessory Cholerae Enterotoxin, pET28a, E. coli

## Abstract

**Objective:**

*Vibrio* cholerae (*V. cholerae*) causes a potentially lethal disease named cholera. The cholera enterotoxin (CT) is a major virulence factor of *V. cholerae*. In addition to CT, *V. cholerae* produces other putative toxins, such as the zonula occludens toxin (Zot) and accessory cholera enterotoxin (Ace). The ace gene is the third gene of the *V. cholerae* virulence cassette. The Ace toxin alters ion transport, causes fluid accumulation in ligated rabbit ileal loops, and is a cause of mild diarrhea. The aim of this study is the cloning and overexpression of the *ace* gene into *Escherichia coli* (*E. coli*) and determination of some characteristics of the recombinant Ace protein.

**Materials and Methods:**

In this experimental study, the ace gene was amplified from *V. cholerae* strain 62013, then cloned in a pET28a expression vector and transformed into an *E. coli* (DH5 α) host strain. Subsequently, the recombinant vector was retransformed into *E. coli* BL21 for expression, induced by isopropythio-β-D-galctoside (IPTG) at a different concentration, and examined by SDS-PAGE and Western blot. A rabbit ileal loop experiment was conducted. Antibacterial activity of the Ace protein was assessed for *E. coli*, Stapylococcus aureus (*S. aureus*), and Pseudomonas aeruginosa (*P. aeruginosa*).

**Results:**

The recombinant Ace protein with a molecular weight of 18 kDa (dimeric form) was expressed in *E. coli* BL21. The Ace protein showed poor staining with Coomassie blue stain, but stained efficiently with silver stain. Western blot analysis showed that the recombinant Ace protein reacted with rabbit anti-*V. cholerae* polyclonal antibody. The Ace protein had antibacterial activity at a concentration of ≥200 µg/ml and caused significant fluid accumulation in the ligated rabbit ileal loop test.

**Conclusion:**

This study described an *E. coli* cloning and expression system *(E. coli BL21- pET-28a-ace)* for the Ace protein of *V. cholerae*. We confirmed the antibacterial properties and enterotoxin activity of the resultant recombinant Ace protein.

## Introduction

*Vibrio* cholerae (V. cholera), is a major human intestinal pathogen that causes significant morbidity and mortality in developing regions of the world. Cholera is endemic in Southern Asia and parts of Africa and Latin America, with 5 million cases annually. This disease is characterized by severe diarrhea caused by toxigenic *V. cholerae* which colonize in the small intestine (1-4).

The cholera toxin (CT) is responsible for severe dehydration that results from diarrhea associated with *V. cholerae*.

*V. cholerae* produces other putative toxins, such as zonula occludens (Zot) and accessory cholera toxin (Ace). The genes encoding these toxins are located on a 4.5 kb region called the "core region" or virulence cassette, which is flanked by two copies of a repeat sequence (1, 2, 5).

The Ace toxin is an integral membrane protein that consists of 96 amino acids (9-11.3 kDa) (6, 7). This toxin increases transcellular ion transport, causing FA in ligated rabbit ileal loops (4). The predicted amino acid sequence of the Ace protein shows a striking similarity to that of a family of eukaryotic ion transporting ATPases, including the human plasma membrane calcium pump, the calcium-transporting ATPase from rat brains, and the product of the cf gene (3, 6). The Ace protein acts synergistically with a Ca^2+^-dependent acetylcholine analog (carbachol) and stimulation secretion has been shown to be dependent on extracellular and intracellular Ca^2+^ (8). The Ace protein also shows a sequence similarity with a virulence protein of *Salmonella dublin*, SpvB, which is virulent in mice (6). Ace is an amphipathic molecule, which when inserted into the eukaryotic cell membrane creates an ion-permeable pore located on the 26-residue δ toxin of *Staphylococcus* aureus (*S. aureus*) (6, 9). This study describes cloning, overexpression of Ace toxins in *Escherichia coli* (*E. coli*), and determination of some characteristics of the recombinant Ace protein.

## Materials and Methods

### Bacterial strains and vectors

In this experimental study, *V. cholerae* strain 62013 was obtained from Pasteur Institute of Iran. *E. coli* DH5α and BL21 (PlysS) were used for cloning and expression experiments (Invitrogen and Novagen, USA). Plasmid *pET-28a^+^* (Novagen) was the expression vector. Bacteria were cultured in LB broth or on agar (Merck, Germany) with or without 30 µg kanamycin/ml (Sigma, USA).

### Preparation of DNA template and PCR

Genomic DNA of the *V. cholerae* strain 62013 was extracted using the Bioneer Kit (South Korea). The concentration and purity of extracted DNA were determined by spectrophotometer. Specific primers were designed according to ace gene sequences of *V. cholerae* from NCBI. The sequence of the forward primer with an endonuclease site of NdeI was 5'-GCTCCATATGCTTATGATGGACACCCTTTATGAC-3', and for the reverse primer with an endonuclease site of EcoRI, it was 5'- TAGAATTCTCATAGGTTTAACGCTCGCAGGGC -3'. The PCR reaction mixture contained 0.5 µM of each primer, 10 µl 5X prime STAR buffer, 0.2 mM of each dNTP, 2.5 U of prime STAR DNA polymerase (Takara, Japan), and 200 ng genomic DNA for a final volume of 50 µl. PCR amplification was performed with an initial denaturation at 98℃ for 4 minutes, followed by 35 cycles at 98℃ for 10 seconds, 63℃ for 15 seconds, and 72℃ for 90 seconds, with a final extension for 10 minutes at 72℃. PCR products were analyzed by electrophoresis on 1% (w/v) agarose gel (Fermentas, USA). The desired fragments were recovered from the gel by a PCR purification kit (Bioneer, South Korea).

### Cloning, expression and purification of Ace

The PCR product and pET-28a (Novagen, United States) expression vector were digested by *NdeI* and EcoRI and purified from in agarose gel. The resultant fragment was ligated by T4 DNA ligase (Fermentas, USA). The recombinant pET-28a was transformed to competent *E. coli* DH5α and the transformants were selected on LB agar plates that contained 30 µg/ml kanamycin.

The selected clones were confirmed by restriction enzyme digestion and PCR, and sequenced by a commercial facility using universal forward and reverse T7-promoter and T7-terminator primers (TAG Copenhagen A/S Symbion, Denmark). The result was compared to the sequence of the *ace* gene in the database with NCBI Blast Software. The recombinant plasmids were retransformed to an *E. coli* BL21 (plysS) expression host. Several conditions for the expression were tested, such as the temperature of induction and the concentration of isopropythio-β-D-galctoside (IPTG). Bacterial cells grew in the presence of kanamycin (30 µg/ml) at 37℃ with shaking (220 rpm) until an optical density at 600 nm of 0.6-1 was reached. IPTG (Sigma, USA) was added to a final concentration of 1 mM, followed by an additional 4 hours culture period at 37℃ with vigorous shaking. Cells were harvested by centrifugation at 10000 g for 10 minutes at 4℃ to precipitate the pellet, after which the pellet was frozen at -20℃.

The bacterial pellets were lysed using a lysis buffer (8 M urea) until the solution cleared. After centrifugation, the supernatants were examined by SDS-PAGE to verify the expressed recombinant protein.

Ace protein was purified by Ni-NTA affinity chromatography under a combination of denaturing and native conditions by binding, washing, and eluting steps according to the manufacturer’s protocol (Invitrogen). In this protocol, proteins were finally eluted in 20 mM buffer that contained imidazole, and then the eluted proteins were immediately dialyzed against PBS (pH= 7.4) for removal of imidazole. Protein concentrations were determined by Bradford and nanodrop analysis and purity by SDS-PAGE. Because Ace is an acidic protein (pI 4.26), it did not stain with standard Coomassie blue staining, thus we used silver nitrate staining according to the standard protocol (2).

### Anti-Ace polyclonal antibody production

The overnight culture of *V. cholerae* 62013 (toxigenic) was exposed to formalin (1.5%) at 4℃ overnight. After washing and deformalization, the culture was heated at 65℃ for 1 hour. The lysate was injected subcutaneously into a white New Zealand rabbit that weighed about 2 kg. The injection mixture contained approximately 109 bacteria per ml of physiological serum and 1 ml complete adjuvant for the first injection, from which 0.5 ml was subcutaneously injected into the shoulder. We used incomplete adjuvant as the booster on days 14, 28, and 42. Bleeding was performed prior to each injection, and the serum was separated and stored at -20℃ until use (10).

### Western blot analysis

The proteins separated by SDS-PAGE were blotted onto 0.45 µm pore size PVDF membrane (Hi-bond Amersham Biosciences, USA) by using a semidry blotter unit (Bio-rad, USA). The membrane was blocked by 3% (w/v) skim milk according to standard procedures. Rabbit polyclonal anti-*V. cholerae* serum was diluted 1:500 in phosphate-buffered saline (PBS) and 0.1% (v/v) Tween 20, then incubated for 3 hours at 4℃ with shaking. The blocked membranes were washed with PBS-Tween 20, and then incubated with affinity purified goat anti-rabbit immunoglobulin G (heavy and light chain) horseradish peroxidase (HRP) conjugate antibody (Bio-Rad), at a 1:2500 dilution in PBS-Tween20. The membranes were then washed three times with PBS-Tween 20 and developed using DAB solution (Sigma, USA) (11).

### Ligated rabbit ileal loop assay

New Zealand rabbits that weighed 2-2.5 kg were starved for 48 hours before the experiments. Rabbits were anesthetized by subcutaneous injection of a mixture of ketamine (50 mg/g) and acepromazine (0.5 mg/kg), and the small intestines were tied off. A total of 1 ml (500 µg/ml) of the purified Ace protein was injected into the two intestinal segments. In this experiment, 1 ml (10^8^ cfu/ml) of *V. cholerae* 62013 was the positive control and sterile PBS was the negative control. Each test was undertaken on two rabbits and all rabbits were sacrificed after 18 hours. The enterotoxic response was determined by measuring the FA ratio, which is the ratio of the volume of fluid accumulated in the intestinal loop to the length of the loop. A ratio of greater than 1.0 is indicative of a strong positive response, while a negative response is defined as a ratio of less than 0.5 (11-16).

### Antibacterial assay

We used bacterial strains *S. aureus* (ATCC25923), Pseudomonas aeruginosa (*P. aeruginosa*; ATCC27853), and *E. coli* (ATCC25922) to assay the antibacterial activity of the Ace protein. Cultures of each bacteria were grown overnight in LB broth, then inoculated into fresh media and grown at 37℃ until the OD595 reached 0.6. The cells were harvested and washed with 10 mM sodium phosphate (pH=7.4), then diluted in media to obtain a final density of 10^3^ cfu/ml per assay sample. Bacteria were incubated with increasing concentrations of Ace protein (0, 50, 100, and 200 µg/ml) at 37℃ for 18 hours in an incubator. The cell growth was determined by colony count (7).

## Results

### Amplification of ace gene and construction of pET28a-ace

**Fig 1:**
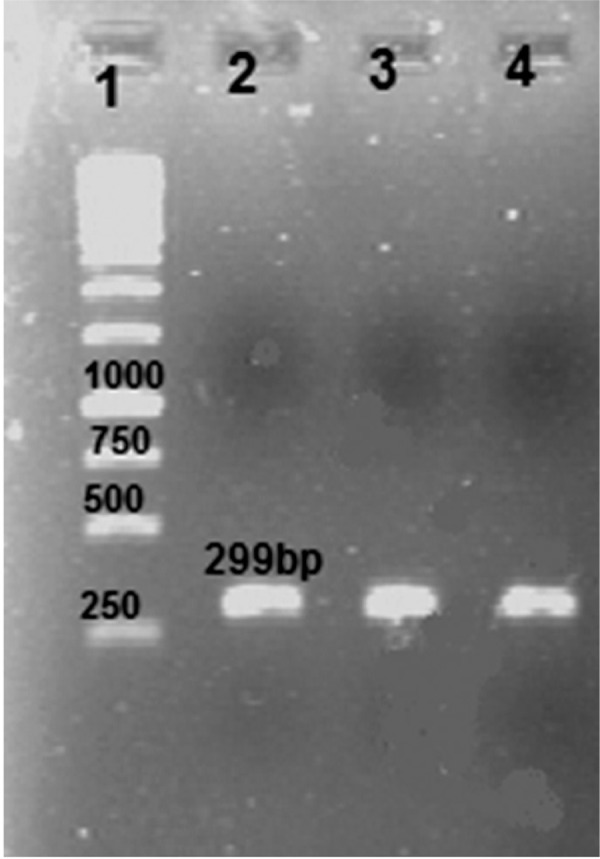
Electrophoresis of ace gene amplified from *V. cholerae* on agarose gel (1% w/v). Lane 1. 1 kb DNA size marker, lanes 2, 3, 4. single expected band of ace (approximately 299 bp).

**Fig 2:**
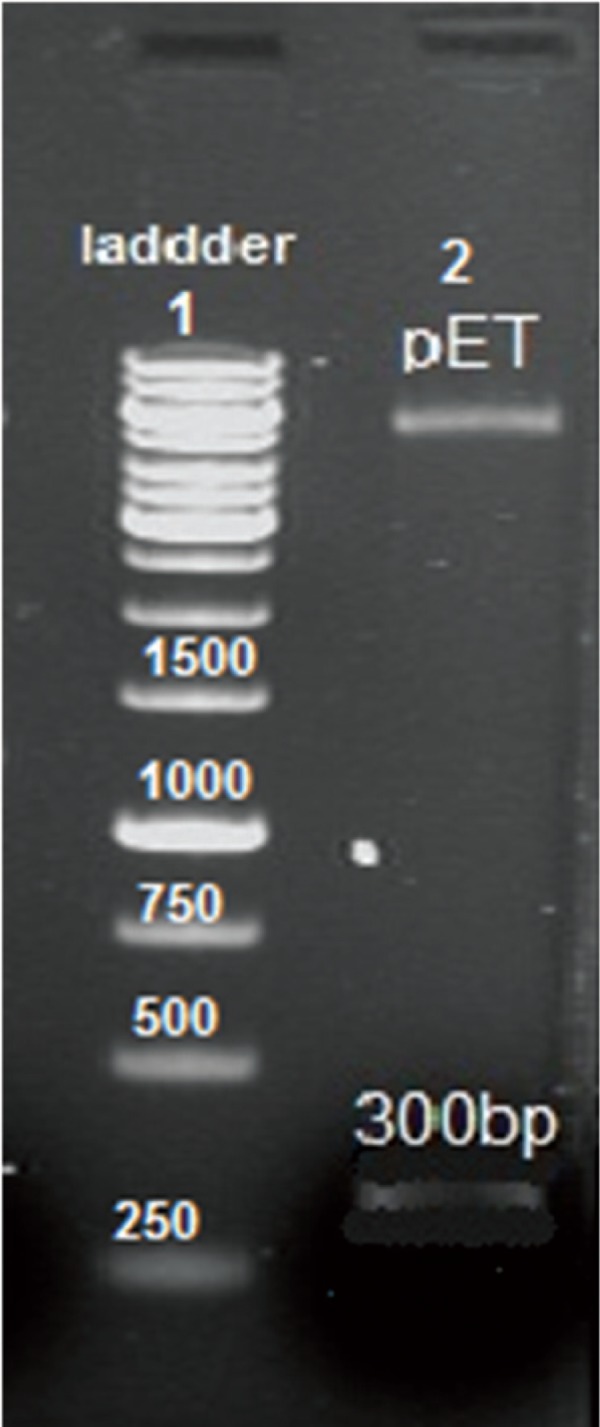
Agarose gel electrophoresis analysis of recombinant pET28a-ace. Lane 1. 1 kb DNA size marker, lane 2. double digestion of recombinant pET28a-ace with EcoRI, NdeI.

The amplified ace gene produced a single 299 bp band (Fig 1). The PCR product was purifi ed and digested with *NdeI* and EcoRI, then subcloned into the expression vector pET28a. The result of the double enzyme digestion and PCR amplifi cation confirmed that the ace gene was exactly inserted into the PET28a vector (Fig 2). The *ace* gene nucleotide sequence in the recombinant plasmid vector of *pET28a-ace* was consistent with that of *V. cholerae* ace as published in the gene bank. The homologies of the nucleotide sequences in the pET-32a-ace compared with the published ace gene sequences were 99.8%.

### Expression and purification of target recombinant protein

**Fig 3:**
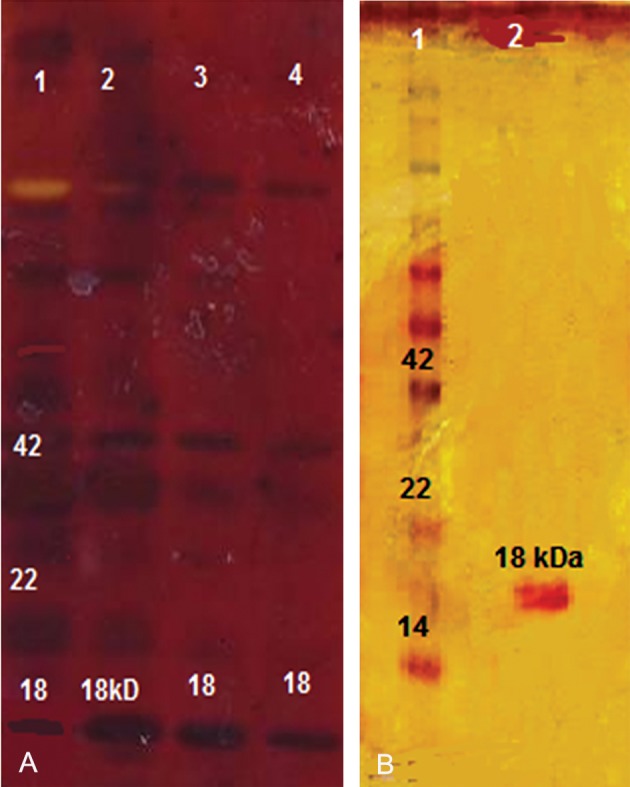
A. SDS-PAGE (15% w/v) analysis of expression product of pET28a-ace in *E. coli* BL21. Lane 1. protein marker, lanes 2, 3, 4. induction of pET28a-ace by treatment with 1mM IPTG (18 kDa). B. recombinant proteins purified by Ni-NTA column chromatograph. Lane 1. protein marker, lane 2. purified rAce protein (18kDa).

*E. coli* BL21 (DE3) plysS competent cells were transformed with the confirmed recombinant vector, *pET28a-ace*. IPTG at 1 mmol/L efficiently induced the expression of *ace* fusion protein with predicated molecular masses of 18 KD (Fig 3A). Ace protein stained poorly with Coomassie blue stain, but stained efficiently with silver stain. Large scale culture and induction was performed and the resultant protein was purified by Ni^2+^ affinity chromatography under denatured and native conditions (Fig 3B).

### Western blot analysis

**Fig 4:**
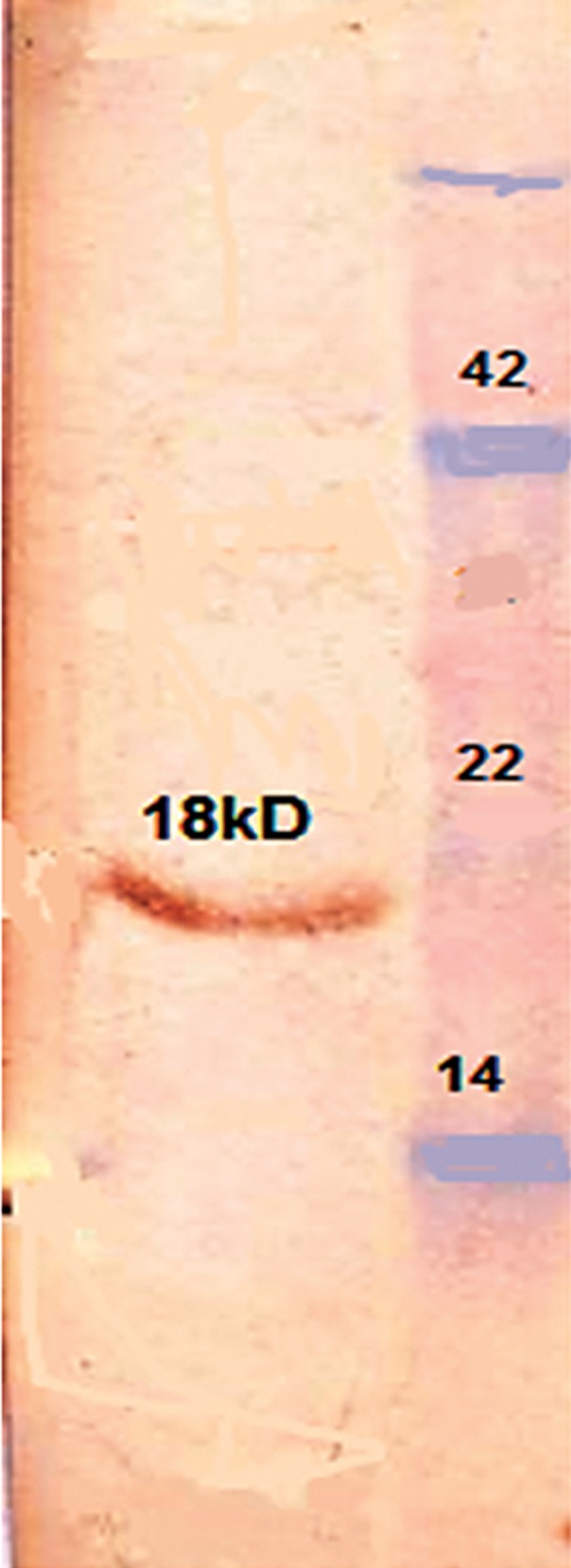
Western blot of the SDS-polyacrylamide gel prepared with anti-*V. cholerae* antibody. Antiserum was diluted 1:500. The 18 kDa proteins of the recombinant Ace were detected.

We performed Western blot analysis to detect immunogenicity of the expressed Ace protein, which was recognized by the rabbit polyclonal antibody against *V. cholerae* (Fig 4).

### Rabbit ileal loop test with recombinant protein

**Fig 5:**
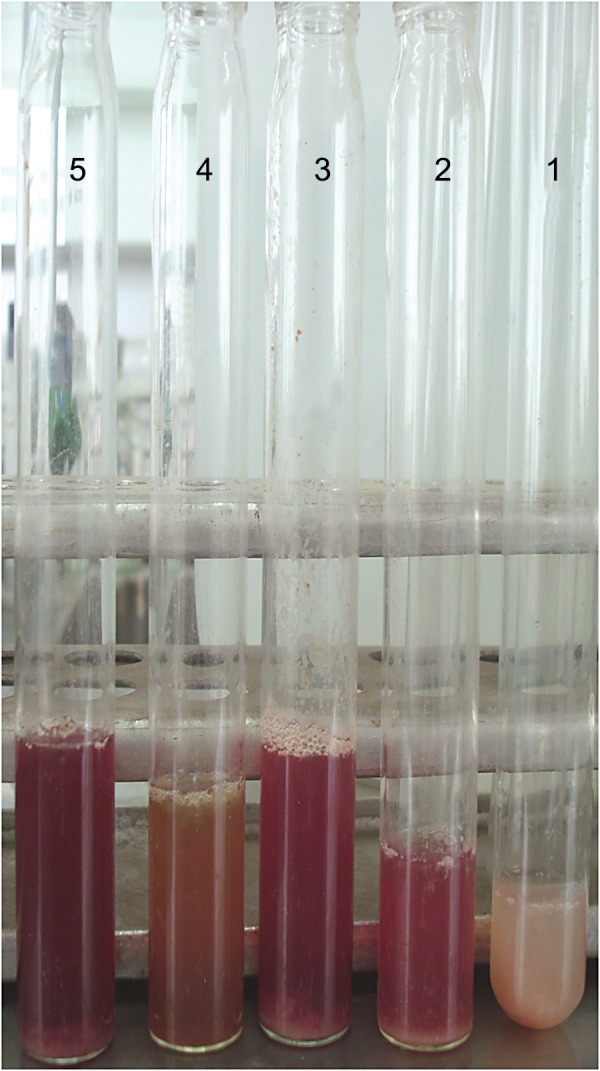
Rabbit ileal loop assay. Ileal tissues treated with recombinant Ace protein culture supernatants of the toxigenic *V. cholerae* 62013 (positive control). Sterile PBS was used as a negative control. Tube 1. fluid accumulation (FA) in negative control (ratio 0.5 ± 0.005), tube 2. significant hemorrhagic FA (ratio 1.25 ± 0.2) with recombinant Ace protein, tubes 3 and 4. other test substances, and tube 5. significant hemorrhagic FA (ratio 2 ± 0.2) with positive control.

The purified Ace protein (500 µg) induced significant FA (ratio: 1.25 ± 0.2) in segmented rabbit ileal loops (Fig 5). FA of PBS (negative control) was not significant (ratio: 0.5 ± 0.005), while FA of the positive control was significant (ratio: 2 ± 0.2).

### Antibacterial assay

We tested for antibacterial activity of purified the recombinant Ace protein to *S. aureus*, *E. coli*, and *P. aeruginosa*. There was inhibition and decreased bacterial growth at the 200 µg/ml concentration of Ace protein.

## Discussion

Ace is a third toxin of *V. cholerae* (1) that causes milder cholera symptoms. It may contribute to an early phase of intestinal secretion in infections by *V. cholerae*, which can occur prior to the onset of secretion stimulated by the cholera toxin (8). Non-enterotoxigenic *V. cholerae* cells that lack *ace, zot*, and ctx genes do not cause diarrhea in volunteers (14). Ace toxin increases the potential difference (PD) across the intestinal epithelium, alters ion transport, and increases the short-circuit current in rabbit ileal tissues that have been mounted in Ussing chambers (1-4, 14, 15). In this study the cloning of the *ace* gene was confirmed by colony-PCR, enzymatic digestion, and sequencing in *E. coli* BL21-pET-28a-*ace*. The nucleotide sequence of the *ace* gene in plasmid pET28a was 100% homologous with the *ace* gene reported in the gene bank ( Z22569.1).

The quantity of Ace protein produced in wild type *V. cholerae* was estimated to be 0.6 mg/lit, which was 10000 fold lower than produced in our expression system (5 mg/lit). The production of the recombinant Ace protein in a concentration of 7 mg/lit in a yeast system was reported previously by Trucksis et al. (2). However, because of *E. coli’s* ability to grow rapidly and at a high density on inexpensive substrates, the prokaryotic expression system has remained very attractive for the production of recombinant proteins (15). According to other studies, expression of the recombinant Ace protein was successful in *E. coli* (LMG194), but in *E. coli* TOP10, the Ace protein was not successfully produced (1, 4).

Ace protein was purified by affinity chromatography with Ni-NTA resin. The predominant form of the Ace toxin was an 18 kD dimeric form. The Ace protein was not completely denatured and persisted as a dimer and multimer in the gel (1). TheAce protein was detected in both monomer and dimeric forms by Trucksis et al. (2). Chatterjee et al. (7) reported only the dimeric forms of this protein. Our recombinant Ace protein did not stain with Coomassie blue, thus we used silver stain, as reported previously (1, 3, 15). The immunogenicity of this protein was confirmed by polyclonal antibody against *V. cholerae*, by enterotoxicity in rabbit ileal loops, and its antimicrobial effects showed it is active biologically. Ace protein has recently been used to treat cystic fibrosis (CF). CF involves insufficient chloride transport and loss of luminal sodium and water, leading to damage of the bronchial connective tissue. The administration of Ace increases the level of secretion of chloride in the lungs and leads to an increase in the amount of airway surface water in the lumen of the lungs (9). Therefore, recombinant production of the Ace protein can be useful for medical applications.

## Conclusion

Our highly efficient expression system *(E. coli BL21- pET-28a-ace)* can be used for conducting various biological experiments and has facilitated the production of pure proteins free from other *Vibrio* antigens for investigation as potential vaccine candidates.
